# Network experiment designs for inferring causal effects under interference

**DOI:** 10.3389/fdata.2023.1128649

**Published:** 2023-04-17

**Authors:** Zahra Fatemi, Elena Zheleva

**Affiliations:** Department of Computer Science, University of Illinois Chicago, Chicago, IL, United States

**Keywords:** causal inference, direct treatment effects, total treatment effects, interference, spillover, selection bias

## Abstract

Current approaches to A/B testing in networks focus on limiting interference, the concern that treatment effects can “spill over” from treatment nodes to control nodes and lead to biased causal effect estimation. In the presence of interference, two main types of causal effects are direct treatment effects and total treatment effects. In this paper, we propose two network experiment designs that increase the accuracy of direct and total effect estimations in network experiments through minimizing interference between treatment and control units. For direct treatment effect estimation, we present a framework that takes advantage of *independent sets* and assigns treatment and control only to a set of non-adjacent nodes in a graph, in order to disentangle peer effects from direct treatment effect estimation. For total treatment effect estimation, our framework combines weighted graph clustering and cluster matching approaches to jointly minimize interference and selection bias. Through a series of simulated experiments on synthetic and real-world network datasets, we show that our designs significantly increase the accuracy of direct and total treatment effect estimation in network experiments.

## 1. Introduction

Causal inference plays a central role in many disciplines, from economics (Varian, 2016; Holtz et al., 2020) to health sciences (Antman et al., 1992; Loucks and Thuma, 2003) and social sciences (Sobel, 2000; Gangl, 2010). The goal of causal inference is to estimate the effect of an intervention on individuals' outcomes. The gold standard for inferring causality is the use of *controlled experiments*, also known as A/B tests and Randomized Controlled Trials (RCTs), in which experimenters can assign treatment (e.g. a news feed ranking algorithm) to a random subset of a population and compare their outcomes with the outcomes of a control group, randomly selected from the same population (e.g., a group of users who used the old news feed ranking algorithm). Through randomization, the experimenter can control for confounding variables that can impact the treatment and outcome assignment but are not present in the data and assess whether the treatment can cause the target variable to change.

While it is straightforward to randomly assign treatment and control to units that are i.i.d., it is much harder to do that for units that interact with each other. The goal of designing *network experiments* is to ensure reliable causal effect estimation in controlled experiments for potentially interacting units. One of the challenges in network experiment design is dealing with interference (or spillover), the problem of treatment “spilling over” from a treated node to a control node. The presence of interference breaks the Stable Unit Treatment Value Assumption (SUTVA), the assumption that one unit's outcome is unaffected by another unit's treatment assignment, and challenges the validity of causal inference (Imbens and Rubin, 2015). Different types of causal estimands are possible in the presence of interference: 1) the difference between the average outcomes of treated and untreated individuals due to the treatment alone (*Direct Treatment Effects*), 2) the influence of peers' behavior on the unit's response to the treatment (*Peer Effects*), and 3) the combination of direct treatment effects and peer effects (*Total Treatment Effects*). Different estimands lead to different inference procedures—both from a design and an analysis point of view. As a motivating example, consider the problem of quantifying the effect of changing the news feed ranking algorithm of an online social network website on the time that users spend interacting with the site. Direct treatment effects capture the effect of changing the news feed ranking algorithm on the time that a user spends on the website, regardless of the behavior of other users in the study. Peer effects quantify the effect of friends time spent on the website on the time that a user spends on the website. Total treatment effects show the total effect of changing the news feed ranking algorithm on the time all users spend on the website which is equal to the sum of peer effects and direct treatment effects.

The focus of this paper is measuring direct and total treatment effects in network data. The total treatment effect of applying a treatment to all units compared with applying a different (control) treatment to all units is a common causal estimand in network experiments. Prominent methods for total treatment effect estimation rely on two-stage or cluster-based randomization, in which clusters are identified using graph clustering and cluster randomization dictates the node assignment to treatment and control (Ugander et al., 2013; Eckles et al., 2016; Saveski et al., 2017; Pouget-Abadie et al., 2018; Fatemi and Zheleva, 2020). Graph clustering aims to find densely connected clusters of nodes, such that few edges exist across clusters (Schaeffer, 2007). The basic idea of applying it to causal inference is that little interference can occur between nodes in different clusters.

**Figure 1:**
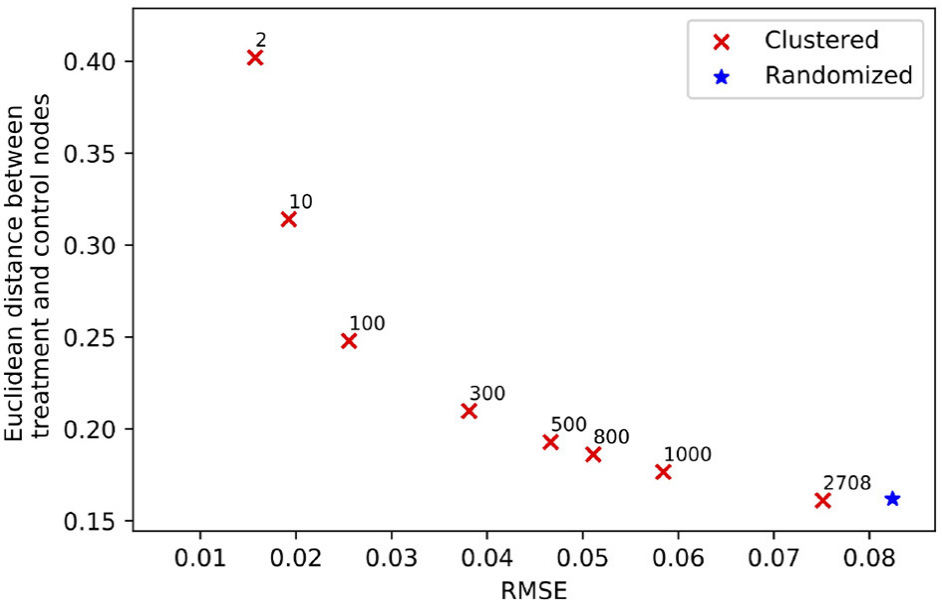
The tradeoff between selection bias (distance) and undesired spillover (RMSE) in cluster-based randomization; each data point is annotated with the number of clusters.

Clustering a connected graph component is guaranteed to leave edges between clusters, therefore removing interference completely is impossible. At the same time, some node pairs are more likely to interact than others, and assigning such pairs to different treatment groups is more likely to lead to undesired spillover (and biased causal effect estimation) than separating pairs with a low probability of interaction. We make the key observation that there is an inherent tradeoff between interference and selection bias in cluster-based randomization based on the chosen number of clusters (as demonstrated in Figure 1). Due to the heterogeneity of real-world graphs, discovered clusters can be very different from each other, and the nodes in these clusters may not represent the same underlying population (Fatemi and Zheleva, 2020). Therefore, cluster randomization can lead to selection bias in the data with causal effects that are confounded by the difference in node features of each cluster.

Here, we propose two methods for network experiment design in the presence of interference. First, we focus on quantifying direct treatment effects by designing a network experiment that disentangles peer effects from direct treatment effects and provides an unbiased estimation of direct treatment effects. We develop *CauseIS*, a framework that leverages independent algorithms on network nodes to divide nodes into two sets: 1) *independent set nodes*, and 2) graph nodes that are not in the independent set referred to as *bystander nodes*. By assigning the independent set nodes to treatment and control groups, we ensure that there are no peer effects between nodes participating in the experiment, regardless of whether they are in different treatment groups or the same treatment group. Key to the proposed experiment design is the idea that in expectation, the peer effects of bystander nodes on the treatment group are the same as the peer effect of bystander nodes on the control group, thus canceling each other in the total treatment effect estimation.

The second method focuses on total treatment effect estimation. We develop *CMatch*, a framework for network experiment design that minimizes both interference and selection bias through a novel objective function for matching clusters and combining node matching with weighted graph clustering to provide a more accurate estimation of total treatment effects (Fatemi and Zheleva, 2020). We introduce the concept of “edge spillover probability” as the probability of interaction between entities and account for it in the design. In this work, incorporating node matching and edge spillover probabilities into graph clustering is novel.

## 2. Related works

By attracting attention toward network experiments, dependent on the assumptions made in the study different causal estimands for direct, peer, and total treatment effects have been proposed (Halloran and Struchiner, 1995; Hudgens and Halloran, 2008; Green et al., 2016; Taylor and Eckles, 2018; Pouget-Abadie et al., 2019; Ugander and Yin, 2020; Aronow et al., 2021; Sävje et al., 2021). In this section, we give an overview of relevant works to quantify direct and total treatment effects in RCTs.

### 2.1. Direct treatment effect estimation

Estimating the effect of treatment alone has been studied in the context of network experiment design. Jagadeesan et al. (2020) propose an approach to reduce the bias of the Neymanian estimator of direct treatment effect estimation under interference and homophily. In this approach, treatment assignment is considered as a quasi-coloring on a graph and every treated node is tried to be matched with a control node with an identical number of treated and control neighbors to create a balanced interference in network experiments. In networks where perfect quasi-coloring is not possible, nodes are ordered by degree and then nodes with a similar degree are paired and assigned to treatment or control. The accuracy of causal effect estimation in this method depends on the network structure, degree distribution of the nodes, and approaching perfect quasi-coloring to perfect quasi-coloring. Recently, Li and Wager (2022) explore the problem of direct treatment effect estimation under random graph asymptotics where an interference graph is a random draw from an (unknown) graphon. Sussman and Airoldi (2017) propose an approach to estimate direct treatment effects considering a fixed design for potential outcomes. Similar to these approaches, we focus on estimating direct treatment effects in the presence of peer effects, but our approach can be applied in networks with different structural properties.

### 2.2. Total treatment effect estimation

Recent work that addresses interference in graphs relies on separating data samples through graph clustering (Backstrom and Kleinberg, 2011; Ugander et al., 2013; Gui et al., 2015; Eckles et al., 2016; Saveski et al., 2017; Pouget-Abadie et al., 2018), relational d-separation (Maier et al., 2010, 2013; Rattigan et al., 2011; Marazopoulou et al., 2015; Lee and Honavar, 2016), or sequential randomization design (Toulis and Kao, 2013). Among these approaches, cluster-based randomization methods attract significant attention recently. Graph clustering aims to find subgraph clusters with high intra-cluster and low inter-cluster edge density (Zhou et al., 2009; Yang and Leskovec, 2015). A number of algorithms exist for weighted graph clustering (Schaeffer, 2007). Node representation learning approaches range from graph motifs (Milo et al., 2002) to embedding representations (Hamilton et al., 2017) and statistical relational learning (SRL) (Rossi et al., 2012). Eckles et al. (2016) evaluate different methods for designing and analyzing randomized experiments and find substantial bias reduction in cluster-based randomization approaches, especially in networks with more clusters and stronger peer effects. Saveski et al. propose a procedure to detect interference bias in network experiments and propose a cluster-based randomization approach to mitigate interference bias in such studies. By comparing completely randomized and Cluster-based randomized experiments (Saveski et al., 2017) on LinkedIn's experimental platform, they indicate the presence of network effects and bias in standard RCTs in a real-world setting. However, cluster-based randomized approaches have high variance, making them more difficult to accurately estimate the treatment effect. Ugander et al. (2013) define a restricted-growth condition on the growth rate of node's connections and show that the variance of estimators is bounded by the linear function of the degrees.

In controlled experiments, the treatment assignment is randomized by the experimenter, whereas in estimating causal effects from observational data, the process by which the treatment is assigned is not decided by the experimenter and is often unknown. Matching is a prominent method for mimicking randomization in observational data by pairing treated units with similar untreated units. Then, the causal effect of interest is estimated based on the matched pairs, rather than the full set of units present in the data, thus reducing the selection bias in observational data (Stuart, 2010). There are two main approaches to matching, fully blocked and propensity score matching (PSM) (Stuart, 2010). Fully blocked matching selects pairs of units whose distance in covariate space is under a pre-determined distance threshold. PSM models the treatment variable based on the observed covariates and matches units that have the same likelihood of treatment. The few research articles that look at the problem of matching for relational domains (Oktay et al., 2010; Arbour et al., 2014) consider SRL data representations. None of them consider cluster matching for a two-stage design which is one of our contributions.

## 3. Preliminaries

In this section, we formally define the data model, the potential outcomes frameworks, and different types of causal estimands.

### 3.1. Data model

A graph *G* = (**V**, **E**) consists of a set of *n* nodes **V** and a set of edges **E** = {*e*_*ij*_} where *e*_*ij*_ denotes that there is an edge between node *v*_*i*_ ∈ **V** and node *v*_*j*_ ∈ **V**. Let **N**_*i*_ denote the set of neighbors for node *v*_*i*_, i.e. set of nodes that share an edge with *v*_*i*_. Let *v*_*i*_.**X** denote the pre-treatment node feature variables (e.g., Twitter user features) for unit *v*_*i*_. Let *v*_*i*_.*Y* denote the outcome variable of interest for each node *v*_*i*_ (e.g., voting), and *v*_*i*_.*T* ∈ {0, 1} denote whether node *v*_*i*_ (e.g., social media user) has been treated (e.g., shown a post about the benefits of voting), *v*_*i*_.*T* = 1, or not, *v*_*i*_.*T* = 0. Let **Z** ∈ {0, 1}^*N*^ be the treatment assignment vector of all nodes. *V*_1_ and *V*_0_ indicate the sets of units in treatment and control groups, respectively. For simplicity, we assume that both *v*_*i*_.*T* and *v*_*i*_.*Y* are binary variables. The edge spillover probability *e*_*ij*_.*p* refers to the probability of interference occurring between two nodes.

### 3.2. Potential outcomes framework

The fundamental problem of causal inference is that we can observe the outcome of a target variable for an individual *v*_*i*_ in either the treatment or control group but not in both. Let *v*_*i*_.*y*(1) and *v*_*i*_.*y*(0) denote the *potential outcomes* of *v*_*i*_.*y* if unit *v*_*i*_ were assigned to the treatment or control group, respectively. The treatment effect (or causal effect) is the difference *g*(*i*) = *v*_*i*_.*y*(1) − *v*_*i*_.*y*(0). Since we can never observe the outcome of a unit under both treatment and control simultaneously, the effect $\hat{\mu }$ of a treatment on an outcome is typically calculated through averaging outcomes over treatment and control groups *via* difference-in-means: $\hat{\mu }=\bar{V_{1}.Y}-\bar{V_{0}.Y}$ (Stuart, 2010). For the treatment effect to be estimable, the following *identifiability* assumptions have to hold:

- *Stable unit treatment value assumption* (SUTVA) refers to the assumption that the outcomes *v*_*i*_.*y*(1) and *v*_*i*_.*y*(0) are independent of the treatment assignment of other units: {*v*_*i*_.*y*(1), *v*_*i*_.*y*(0)}⊥*v*_*j*_.*T*, ∀*v*_*j*_ ≠ *v*_*i*_ ∈ *V*.
- *Ignorability* (Imbens and Rubin, 2015)—also known as *conditional independence* (Pearl, 2009) and *absence of unmeasured confoundness*—is the assumption that all variables *v*_*i*_.*X* that can influence both the treatment and outcome *v*_*i*_.*Y* are observed in the data and there are no unmeasured confounding variables that can cause changes in both the treatment and the outcome: {*v*_*i*_.*y*(1), *v*_*i*_.*y*(0)}⊥*v*_*i*_.*T*∣*v*_*i*_.*X*.
- *Overlap* is the assumption that each unit assigned to the treatment or control group could have been assigned to the other group. This is also known as *positivity* assumption: *P*(*v*_*i*_.*T*|*v*_*i*_.*X*) > 0 for all units and all possible *T* and *X*.

### 3.3. Types of causal effects in networks

We follow Hudgens and Halloran (2008) to define causal estimands for different types of effects possible in the presence of interference. However, our setting is different in a way that all nodes in the same group receive a similar treatment.

*Total Treatment Effects (TTE)* is defined as the outcome difference between two alternative universes, one in which all nodes are assigned to treatment ($Z_{1}={\left\{1\right\}}^{N}$) and one in which all nodes are assigned to control ($Z_{0}={\left\{0\right\}}^{N}$) (Ugander et al., 2013; Saveski et al., 2017):


$$TTE=\frac{1}{N}\sum_{v_{i}\in V}\left(v_{i}.Y(Z_{1})-v_{i}.Y(Z_{0})\right).$$

*TTE* is estimated as averages over the treatment and control group, and it accounts for two types of effects, *Direct Treatment Effects (DTE)* and *Peer Effects (PE)*:


(1) $$T\hat{T}E=\bar{V_{1}.Y}-\bar{V_{0}.Y}=DTE(V)+PE(V_{1})-PE(V_{0}).$$

Direct Treatment effects (*DTE*) reflects the difference between the outcomes of treated and untreated subjects which can be attributed to the treatment alone. They are estimated as:


(2) $$\begin{array}{l} DTE\left(V\right)=\underset{v_{i}\in V}{𝔼}\left[v_{i}.Y|v_{i}.T=1\right]-\underset{v_{i}\in V}{𝔼}\left[v_{i}.Y|v_{i}.T=0\right]. \end{array}$$

Peer effects (*PE*), known also as indirect effects in the prior studies (Halloran and Struchiner, 1995; Hudgens and Halloran, 2008; Jagadeesan et al., 2020), reflect the difference in outcomes that can be attributed to the influence of other subjects in the experiment. Let *N*_*i*_.***π*** denote the vector of treatment assignments to node *v*_*i*_'s neighbors *N*_*i*_. Average *PE* is estimated as having neighbors with a treatment vector:


(3) $$\begin{array}{l} PE\left(V\right)=\underset{v_{i}\in V}{𝔼}\left[v_{i}.Y|v_{i}.T=t,N_{i}.\pi \right]-\underset{v_{i}\in V}{𝔼}\left[v_{i}.Y|v_{i}.T=t,N_{i}=\emptyset \right]. \end{array}$$

Here, we distinguish between two types of peer effects, *allowable peer effects* (*APE*) and *unallowable peer effects* (*UPE*). Allowable peer effects are peer effects that occur within the same treatment group, and they are a natural consequence of network interactions. For example, if a social media company wants to introduce a new feature (e.g., nudging users to vote), it would introduce that feature to all users and the total effect of the feature would include both individual and peer effects. Unallowable peer effects are peer effects that occur across treatment groups and contribute to undesired spillover and incorrect causal effect estimation.

For each node *v*_*i*_ in treatment group *t*, we have two types of neighbors: 1) neighbors $N_{i}^{t}$ in the same treatment class as node *v*_*i*_ with treatment assignment set $N_{i}^{t}.\pi$; 2) set of neighbors in a different treatment class $N_{i}^{\bar{t}}$ ($\bar{t}\neq t$) with treatment assignment denoted by $N_{i}^{\bar{t}}.\pi$. The *APE* is defined as:


(4) $$\begin{array}{l} APE\left(V\right)=\underset{v_{i}\in V}{𝔼}\left[v_{i}.Y|v_{i}.T=t,N_{i}^{t}.\pi \right]-\underset{v_{i}\in V}{𝔼}\left[v_{i}.Y|v_{i}.T=t,N_{i}^{t}=\emptyset \right], \end{array}$$

and the *UPE* is defined as:


(5) $$\begin{array}{l} UPE\left(V\right)=\underset{v_{i}\in V}{𝔼}\left[v_{i}.Y|v_{i}.T=t,N_{i}^{\bar{t}}.\pi \right]-\underset{v_{i}\in V}{𝔼}\left[v_{i}.Y|v_{i}.T=t,N_{i}^{\bar{t}}=\emptyset \right]. \end{array}$$

## 4. Problem statement

The goal of designing network experiments is to ensure reliable causal effect estimation in controlled experiments by minimizing both unallowable peer effects in node assignment to treatment and control. In this work, we are interested to design two network experiments for quantifying direct and total treatment effects.

### 4.1. Direct treatment effect estimation

The question we are interested to answer is: What is the causal effect of the treatment alone? This question has many practical applications for estimating the effectiveness of different policy interventions. Some examples include: What is the individual protection from a disease due to vaccination alone (and not herd immunity)? What is the effect of advertisements on motivating a person to buy a new phone? In network experiments, it is challenging to disentangle *DTE* from *PE* and this is one of the main goals of this paper. More formally:

 Problem 1 (Network experiment design for direct treatment effect). estimation. Given an undirected graph *G* = (**V**, **E**), and a set of attributes **V.X** associated with each node. Find a treatment assignment vector **Z** of a population with three different subsets of nodes, the treatment nodes **V**_1_ ∈ **V**, the control nodes **V**_0_ ∈ **V**, and nodes excluded from the experiment **V**_2_ ∈ **V**, such that:

a. **V**_0_ ∩ **V**_1_ ∩ **V**_2_ = ∅.
b. |**V**_0_| + |**V**_1_| is maximized.
c. *PE*(*V*_1_) − *PE*(*V*_0_) ≈ 0.

The first component aims to choose treatment, control, and bystander nodes excluded from the experiments that do not overlap. The second component ensures to choose of as many nodes as possible from **V** to be assigned to treatment and control groups. The third component removes peer effects from causal effect estimation.

### 4.2. Total treatment effect estimation

TTE is one of the most popular causal estimands in network experiments, especially in cluster-based randomization approaches (Eckles et al., 2016; Pouget-Abadie et al., 2018). There are two main challenges with causal effect estimation in graphs.

#### 4.2.1. Challenge no. 1: it is hard to separate a graph into treatment and control nodes without leaving edges across

The presence of interference breaks the SUTVA assumption and leads to biased causal effect estimation in relational data. The two-stage experimental design addresses this problem by finding groups of units that are unlikely to interact with each other (stage 1) and then randomly assigning each group to treatment and control (stage 2). Clustering has been proposed as a way to discover such groups that are strongly connected within but loosely connected across, thus finding treatment and control subgraphs that have a low probability of spillover from one to the other. However, due to the density of real-world graphs, graph clustering techniques can leave as many as 65% to 79% of edges as inter-cluster edges (Table 2 in Saveski et al., 2017). Leaving these edges across treatment and control nodes would lead to a large amount of spillover. Incorporating information about the edge probability of spillover into the clustering helps alleviate this problem and is one of the main contributions of our work.

#### 4.2.2. Challenge no. 2: there is a tradeoff between interference and selection bias in cluster-based network experiments

While randomization of i.i.d. units in controlled experiments can guarantee ignorability and overlap, the two-stage design does not. One of the key observations in our work is that dependent on the number of clusters, there is a tradeoff between interference and selection bias in terms of the treatment and control group not representing the same underlying distribution. Figure 1 illustrates this tradeoff for Cora, one of the datasets in our experiments, using *reLDG* as the clustering method. When a network is separated into very few clusters, the Euclidean distance between nodes in treatment and control clusters is larger than the Euclidean distance when a lot of clusters are produced over the same network (e.g., 0.4 vs. 0.18 for 2 and 1, 000 clusters). This is intuitive because as the clusters get smaller and smaller, their randomization gets closer to mimicking full node randomization (shown as a star). At the same time, a larger number of clusters translates to a higher likelihood of edges between treatment and control nodes, which leads to higher undesired spillover and causal effect estimation error (e.g., 0.015 vs. 0.059 for 2 and 1000 clusters).

Ideally, we would like to measure *TTE* = *DTE*(*V*) + *APE*(*V*_1_) − *APE*(*V*_0_). Due to undesired spillover in a controlled experiment, what we are able to measure instead is the overall effect that comprises both allowable and unallowable peer effects *TTE* = *DTE*(*V*) + *APE*(*V*_1_) − *APE*(*V*_0_) + *UPE*(*V*_1_) − *UPE*(*V*_0_). Therefore, when we design an experiment for minimum interference, we are interested in setting it up in a way that makes *UPE*(*V*_1_) = 0 and *UPE*(*V*_0_) = 0. More formally:

Problem 2 (Network experiment design for total treatment effect estimation). Given a graph *G* = (**V**, **E**), a set of attributes **V.X** associated with each node and a set of spillover probabilities **E.P** associated with the graph edges, we want to construct two sets of nodes, the control nodes **V**_0_ ∈ **V** and the treatment nodes **V**_1_ ∈ **V** such that:

a. **V**_0_ ∩ **V**_1_ = ∅.
b. |**V**_0_| + |**V**_1_| is maximized.
c. θ = *UPE*(**V**_1_) − *UPE*(**V**_0_) is minimized.
d. **V**_0_**.X** and **V**_1_**.X** are identically distributed.

This problem definition describes the desired qualities of the experiment design at a high level. The first component ensures that the treatment and control nodes do not overlap. The second component aims to keep as many nodes as possible from **V** in the final design. The third component minimizes unallowable spillover. The fourth component requires that there is no selection bias between the treatment and control groups. The second and third components are at odds with one another and require a tradeoff because the lower θ, the lower the number of selected nodes for the experiment |**V**_0_| + |**V**_1_|. As we showed in Figure 1, there is also a tradeoff between the third and fourth components.

## 5. *CauseIS*: a network experiment design framework for direct treatment effect estimation

In this section, we define an objective function corresponding to the problem of this paper and describe our proposed framework which we refer to as *CauseIS* for estimating direct treatment effects in network experiments.

Typically, total treatment effect estimation includes both APE and UPE. In a randomized approach, TTE is estimated as:


(6) $$\begin{array}{l} T\hat{T}E\left(\text{V}\right)=DTE\left(\text{V}\right)+\left(APE\left({\text{V}}_{1}\right)-APE\left({\text{V}}_{0}\right)\right)+\left(UPE\left({\text{V}}_{1}\right)-UPE\left({\text{V}}_{0}\right)\right). \end{array}$$

In this work, we propose an approach that makes APE(**V**_1_)=0 and APE(**V**_0_)=0 and in expectation makes UPE(**V**_1_)-UPE(**V**_0_)=0, thus making the estimated TTE correspond to DTE. We first define an objective function that addresses the goals specified in *Problem 1*.

### 5.1. Objective function

The goal of the objective function is to find a subset of **V** with maximum cardinality (*Problem 1.b*) such that by randomizing treatment assignment over the selected subset, the allowable peer effects from the experiment are removed (*Problem 1.c*). We define *s* ∈ {0, 1} such that *s*_*i*_ = 1 if node *v*_*i*_ is in the set of selected nodes, else *s*_*i*_ = 0.


$$\begin{array}{l} \text{maximize }\sum_{i=1}^{\left|V\right|}s_{i}\\ \text{subject to }s_{i}+s_{j}\leq 1\;\forall e_{i,j}\in E\\ \;s_{i}\in \{0,1\}\;\forall v_{i}\in V \end{array}$$

The two constraints together guarantee that adjacent nodes are not included in our network experiment design. This optimization can be solved by reducing our problem to the maximum independent set problem in graph theory (Eisenbrand et al., 2003) such that nodes in the independent set correspond to the nodes selected for the network experiment.

Given a graph *G* = (**V**, **E**), **IS** ⊆ **V** is a subset of nodes such that for each pair of nodes *v*_*i*_ ∈ **IS** and *v*_*j*_ ∈ **IS** there is no shared edge between them (*e*_*i,j*_ ∉ **E**). A *maximal independent set* is an independent set that is not a subset of any other independent sets of the graph. Using a greedy sequential approach, a maximal independent set of a graph can be found in *O*(|*E*|) (Blelloch et al., 2012) but there are parallel algorithms that can solve this problem much faster in *O*(*log*(*N*)) (Luby, 1985; Yves et al., 2009). A maximal independent set with the largest possible size for a given graph is known as a *maximum independent set*. Finding maximum independent sets in graphs is known to be NP-hard. There are exact algorithms that can find maximum independent sets in *O*(1.1996^*n*^*n*^*O*(1)^) (Xiao and Nagamochi, 2017) and also approximation algorithms that can find it in *O*(*n*/(*logn*)^2^) (Boppana and Halldórsson, 1990).

### 5.2. *CauseIS* Framework

**Figure 2:**
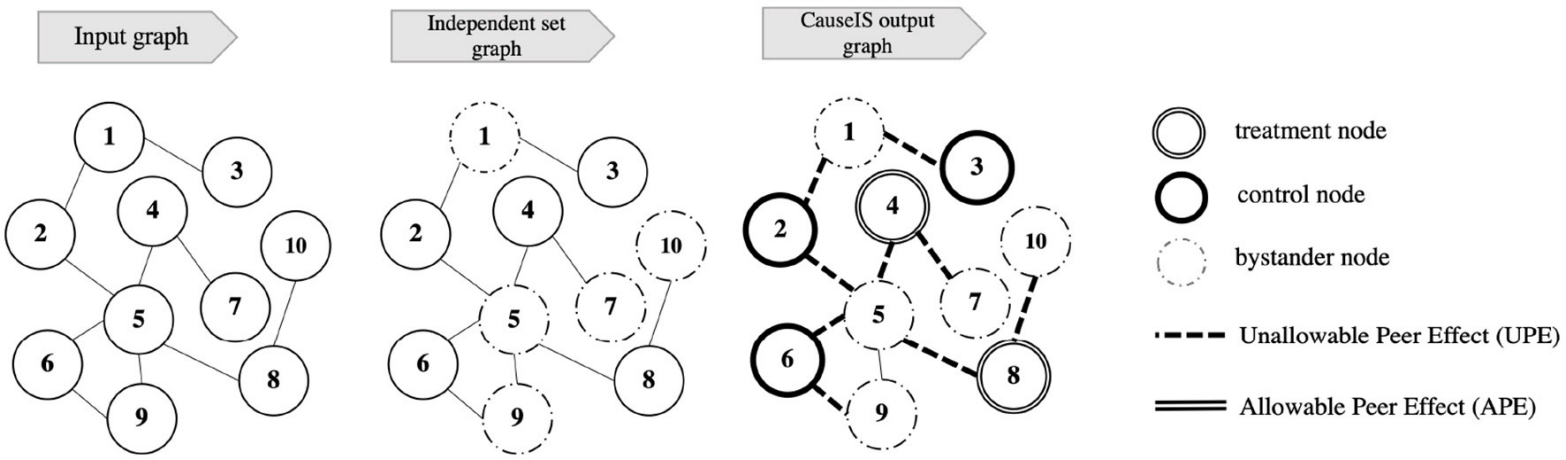
Illustration of *CauseIS* frameworks in network experiments. **Input graph**: a graph of nodes and the connection between them. **Independent set graph**: a graph of bystander and independent set nodes selected by the independent set algorithm. ***CauseIS*** **output graph**: the output graph that represents randomized treatment assignment of independent set nodes and peer effects that exists in the experiment.

We propose *CauseIS*, a network experiment design for robust estimation of Direct Treatment Effects by disentangling peer effects from DTE. *CauseIS* has two main steps:

1. Finding a maximum independent set of the graph (*Independent set graph* in Figure 2).
2. Assigning nodes of the maximum independent set to treatment and control in a randomized fashion (*CauseIS* *output graph* in Figure 2).

In this framework, we find the treatment assignment vector **Z** of nodes by dividing the population into treatment, control, and bystander nodes. Considering the proposed objective function, we first use an algorithm to find the maximum independent set of the given graph which partitions the graph into two sets of nodes: 1) nodes in the maximum independent set denoted by *MIS* (*MIS* ⊆ **V**) where by randomizing treatment assignment over these nodes, we achieve treatment (**V_1_**) and control (**V_0_**) groups, and 2) bystander nodes (**V_2_**) that are not in *MIS* where **V_2_** ⊆ **V**, **V_2_** ∩ **MIS** = ∅, and **V_2_** ∪ **MIS** = **V**. The main idea is to assign nodes of *MIS* to treatment and control at random and ensure that there is no peer effect across treatment and control nodes.

Figure 2 represents the pipeline of the *CauseIS* framework. *Input graph* shows the graph of the network that the network experiment is conducted on. After using an independent set algorithm on the Input graph, independent set and bystander nodes are selected from the graph that is shown in *Independent set graph*. Finally, by randomizing treatment assignment over independent set nodes, treatment, and control nodes are selected. *CauseIS* *output graph* shows the assignment of Input graph nodes to three treatment groups where APE is removed from the experiment.

We remove bystander nodes from the randomized treatment assignment because of the interaction within these nodes which leads to APE in treatment effect estimation. However, it is still possible that information flows from peers in **V_2_** to **V**_0_ and **V**_1_, leading to undesired peer effects (nodes 1, 5, 7, 9, 10 in Figure 2). In the running example, an infected person in **V_2_** may infect his peers in **V**_0_ and **V**_1_.

By removing APE from Equation (6), we have $T\hat{T}E\left(\text{V}\right)=DTE\left(\text{V}\right)+\left(UPE\left({\text{V}}_{1}\right)-UPE\left({\text{V}}_{0}\right)\right)$. By randomizing the treatment assignment over *MIS* nodes, we aim to provide a chance for treatment and control nodes to have the same number of peers in bystander nodes **V_2_**. Let *α*_1_ be the set of bystander nodes that are activated neighbors of treatment nodes, and *α*_2_ be the set of bystander nodes that are activated neighbors of control nodes at time t-1. Let *V*_1,*α*_ and *V*_0,*α*_ represent the set of treatment and control nodes activated by *α*_1_ and *α*_0_ at time t, and let *V*_1,−*α*_ and *V*_0,−*α*_ denote the set of treatment and control nodes not activated by bystander nodes, respectively. Through randomization over set *MIS*, we obtain |*α*_1_| ≈ |*α*_0_|. In this setup, TTE can be estimated as:


(7) $$\begin{array}{l} T\hat{T}E=\left(\frac{1}{\left|{\text{V}}_{1}\right|}\underset{v_{i}\in {\text{V}}_{1\text{,−}\alpha }}{\sum }v_{i}.Y-\frac{1}{\left|{\text{V}}_{0}\right|}\underset{v_{i}\in {\text{V}}_{0\text{,−}\alpha }}{\sum }v_{i}.Y\right)\\ \;+\left(\frac{1}{\left|{\text{V}}_{1}\right|}\underset{v_{i}\in {\text{V}}_{1,\alpha }}{\sum }v_{i}.Y-\frac{1}{\left|{\text{V}}_{0}\right|}\underset{v_{i}\in {\text{V}}_{0,\alpha }}{\sum }v_{i}.Y\right). \end{array}$$

If the probability of activating a treatment and a control node by a bystander node is equal, then, in expectation, an equal number of nodes in treatment and control nodes would get activated by bystander nodes (|*V*_1,*α*_| ≈ |*V*_0,*α*_|) and UPE(V1) is equal to UPE(V0), i.e., $\frac{1}{\left|{\text{V}}_{1}\right|}\underset{v_{i}\in {\text{V}}_{1,\alpha }}{\sum }v_{i}.Y\approx \frac{1}{\left|{\text{V}}_{0}\right|}\underset{v_{i}\in {\text{V}}_{0,\alpha }}{\sum }v_{i}.Y$. As a result, we have:


(8) $$\begin{array}{l} T\hat{T}E=\frac{1}{\left|{\text{V}}_{1}\right|}\underset{v_{i}\in {\text{V}}_{1\text{,−}\alpha }}{\sum }v_{i}.Y-\frac{1}{\left|{\text{V}}_{0}\right|}\underset{v_{i}\in {\text{V}}_{0,-\alpha }}{\sum }v_{i}.Y. \end{array}$$

Since by design there are no peer effects between the treatment and control groups, Equation (8) estimates the DTE (*TTE* ≈ *DTE*).

## 6. *CMatch*: a network experiment design framework for total treatment effect estimation

In this section, we describe our proposed *CMatch* framework that increases the accuracy of TTE by combining clustering and matching techniques.

### 6.1. *CMatch* framework

**Figure 3:**
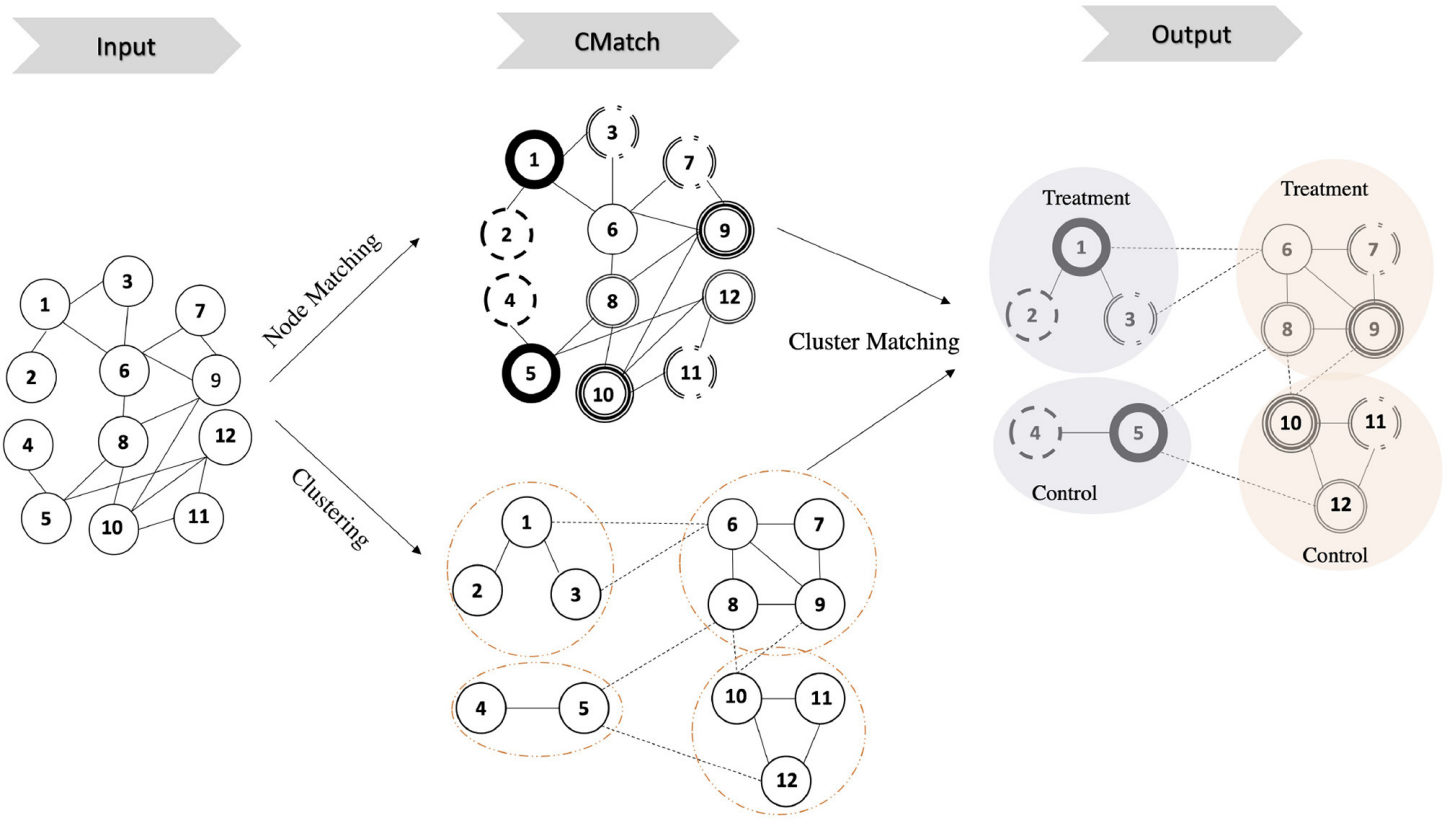
Illustration of *CMatch* framework for minimizing interference and selection bias in controlled experiments. **Input**: a graph of nodes and the connection between them. **CMatch**: node and cluster matching; the dashed circles indicates the clusters. Matched nodes are represented with a similar circle border. **Output**: assigning the matched cluster pairs to treatment and control randomly; circles with the same color represent matched clusters.

Our network experiment design framework *CMatch*, illustrated in Figure 3, has two main goals: 1) *spillover minimization* which it achieves through weighted graph clustering, and 2) *selection bias minimization* which it achieves through cluster matching. Clusters in each matched pair are assigned to different treatments, thus achieving covariate balance between treatment and control (Fatemi and Zheleva, 2020). The first goal addresses part *c* of *Problem 1* and the second goal addresses part *d*. While the first goal can be achieved with existing graph mining algorithms, solving for the second one requires developing novel approaches. To achieve the second goal, we propose an objective function, which can be solved with maximum weighted matching, and present the nuances of operationalizing each step.

#### 6.1.1. Step 1: interference minimization through weighted graph clustering

Existing cluster-based techniques for network experiment design assume unweighted graphs (Backstrom and Kleinberg, 2011; Ugander et al., 2013; Gui et al., 2015; Eckles et al., 2016; Saveski et al., 2017) and do not consider that different edges can have different likelihood of spillover. Incorporating information about the edge probability of spillover into the clustering helps alleviate this problem and is one of the main contributions of our work. In order to minimize undesired spillover, we operationalize minimizing θ as minimizing the edges, and more specifically the edge spillover probabilities, between treatment and control nodes: $\hat{\theta }=\underset{\forall v_{i}\in {\text{V}}_{0},\forall v_{j}\in {\text{V}}_{1}}{\sum }e_{ij}.p$. To achieve this, *CMatch* creates graph clusters for two-stage design by employing two functions, edge spillover probability estimation and weighted graph clustering.

##### 6.1.1.1. Edge spillover probability estimation

We consider edge strength, how strong the relationship between two nodes is, as a proxy for edge spillover probability. This reflects the notion that the probability of a person influencing a close friend to do something is higher than the probability of influencing an acquaintance. We can use common graph mining techniques to calculate edge strength, including ones based on topological proximity (Liben-Nowell and Kleinberg, 2007), supervised classification (Gilbert and Karahalios, 2009), or latent variable models (Li et al., 2010).

##### 6.1.1.2. Weighted graph clustering

In order to incorporate edge strength into clustering, we can use any existing weighted graph clustering algorithm (Enright et al., 2002; Schaeffer, 2007; Yang and Leskovec, 2015). In our experiments, we use a prominent non-parametric algorithm, the *Markov Clustering Algorithm (MCL)* (Enright et al., 2002) which applies the idea of random walk for clustering graphs and produces non-overlapping clusters. We also compare this algorithm with *reLDG* which was the basis of previous work (Saveski et al., 2017). One of the advantages of *MCL* is that it automatically finds the optimal number of clusters, rather than requiring it as input. The main idea behind *MCL* is that nodes in the same cluster are connected with higher-weighted shortest paths than nodes in different clusters.

#### 6.1.2. Step 2: selection bias minimization through cluster matching

Randomizing treatment assignment over clusters in a two-stage design does not guarantee that nodes within those clusters would represent random samples of the population. We propose to address this selection bias problem by *cluster matching* and balancing covariates across treatment and control clusters. While methods for matching nodes exist (Oktay et al., 2010; Stuart, 2010; Arbour et al., 2014), this work is the first to propose methods for matching clusters.

##### 6.1.2.1. Objective function

The goal of cluster matching is to find pairs of clusters with similar node covariate distributions and assign them to different treatment groups. We propose to capture this through a maximum weighted matching objective over a cluster graph in which each discovered cluster from step 1 is a node and edges between clusters represent their similarity. Suppose that graph *G* is partitioned into *C* = {*c*_1_, *c*_2_, ..., *c*_*g*_} clusters. We define *A* ∈ {0, 1}, such that *a*_*ij*_ = 1 if two clusters *c*_*i*_ and *c*_*j*_ are matched, else *a*_*ij*_ = 0. *w*_*i, j*_ ∈ ℝ represents the similarity between two clusters *c*_*i*_ and *c*_*j*_. Then the objective function of *CMatch* is as follows:


(9) $$\begin{array}{l} \underset{A}{argmax}\;\overset{g}{\underset{i=1}{\sum }}\overset{g}{\underset{j=i+1}{\sum }}\left(a_{ij}\cdot w_{ij}\right)\\ \text{subject to }\forall c_{i}\in C,\overset{\left|c_{i}\right|}{\underset{j=1}{\sum }}a_{ij}\leq 1,a_{ij}\in \left\{0,1\right\}. \end{array}$$

This objective function maps to a maximum weighted matching problem for which there is a linear-time approximation algorithm (Duan and Pettie, 2014) and a polynomial-time exact algorithm with *O*(*N*^2.376^) (Mucha and Sankowski, 2004; Harvey, 2009).

##### 6.1.2.2. Solution

In order to operationalize the solution to this objective, the main question that needs to be addressed is: what does it mean for two clusters to be similar? We propose to capture this cluster similarity through matched nodes. The more nodes can be matched based on their covariates across two clusters, the more similar the two clusters are. Thus, the operationalization comes down to the following three questions which we address next:

1. What constitutes a ***node match***?
2. How are node matches taken into consideration in computing the pairwise ***cluster*** ***weights*** (cluster similarity)?
3. Given a cluster weight, what constitutes a potential cluster match, and thus an edge in the ***cluster graph***?

Once these three questions are addressed, the cluster graph can be built and an existing maximum weighted matching algorithm can be applied to it to find the final cluster matches.

###### 6.1.2.2.1. Node Matching

The goal of node matching is to reduce the imbalance between treatment and control groups due to their different feature distributions. Given a node representation, fully blocked matching would look for the most similar nodes based on that representation (Stuart, 2010). It is important to note that propensity score matching does not apply here because it models the probability of treatment in observational data and treatment is unknown at the time of designing a controlled experiment. In its simplest form, a node can be represented as a vector of attributes, including node-specific attributes, such as demographic characteristics, and structural attributes, such as node degree. For any two nodes, it is possible to apply an appropriate similarity measure *sim*(*v*_*i*_, *v*_*j*_), in order to match two nodes, including cosine similarity, Jaccard similarity, or Euclidean distance.

We consider two different options to match a pair of nodes in different clusters (and ignore matches within the same cluster):

- **Threshold-based node matching (TNM)**: Node *v*_*k*_ in cluster *c*_*i*_ is matched with node *v*_*l*_ from a different cluster *c*_*j*_ if the pairwise similarity of nodes *sim*(*v*_*k*_, *v*_*l*_) > *α*. The threshold *α* can vary from 0, which liberally matches all pairs of nodes, to the maximum possible similarity which matches nodes only if they are exactly the same. In our experiments, we set *α* based on the covariate distribution of each dataset and consider different quartiles of pairwise similarity as thresholds. This allows for each node to have multiple possible matches across clusters.
- **Best node matching (BNM)**: Node *v*_*k*_ in cluster *c*_*i*_ is matched with only one node *v*_*l*_ which is most similar to *v*_*k*_ in the whole graph; *v*_*l*_ should be in a different cluster. This is a very conservative matching approach in which each node is uniquely matched but allows the matching to be asymmetric.

###### 6.1.2.2.2. Cluster Weights

After the selection of a node matching mechanism, we are ready to define the pairwise similarity of clusters which is the basis of cluster matching. We consider three simple approaches and three more expensive approaches which require maximum weighted matching between nodes:

- **Euclidean distance (E)**: This approach is the simplest of all because it does not consider node matches and it simply calculates the Euclidean distance between the node attribute vector means of two clusters.
- **Matched node count (C)**: The first approach counts the number of matched nodes in each pair of clusters *c*_*i*_ and *c*_*j*_ and considers the count as the clusters' pairwise similarity: $w_{ij}=\overset{\left|c_{i}\right|}{\underset{k=1}{\sum }}\overset{\left|c_{j}\right|}{\underset{l=1}{\sum }}r_{kl}^{ij}$. A node in cluster *c*_*i*_ can have multiple matched nodes in *c*_*j*_.
- **Matched node average similarity (S)**: Instead of the count, this approach considers the average similarity between matched nodes across two clusters *c*_*i*_ and *c*_*j*_:
  $w_{ij}=\frac{\overset{\left|c_{i}\right|}{\underset{k=1}{\sum }}\overset{\left|c_{j}\right|}{\underset{l=1}{\sum }}r_{kl}^{ij}\cdot sim\left(v_{k},v_{l}\right)}{\overset{\left|c_{i}\right|}{\underset{k=1}{\sum }}\overset{\left|c_{j}\right|}{\underset{l=1}{\sum }}r_{kl}^{ij}}$.

These first two approaches allow a single node to be matched with multiple nodes in another cluster and each of those matches counts toward the cluster pair weight. In order to distinguish this from a more desirable case in which multiple nodes in one cluster are matched to multiple nodes in another cluster, we propose approaches that allow each node to be considered only once in the matches that count toward the weight. For each pair of clusters, we build a node graph in which an edge is formed between nodes *v*_*i*_ and *v*_*j*_ in the two clusters and the weight of this edge is *sim*(*v*_*i*_, *v*_*j*_). Maximum weighted matching will find the best possible node matches in the two clusters. We consider three different variants for calculating the cluster pair weight based on the maximum weighted matching of nodes:

- **Maximum matched node count (MC)**: This method calculates the cluster weight the same way as **C** except that the matches (whether $r_{kl}^{ij}$ is 0 or 1) are based on the maximum weighted matching result.
- **Maximum matched node average similarity (MS)**: This method calculates the cluster weight the same way as **S** except that the node matches are based on the maximum weighted matching result.
- **Maximum matched node similarity sum (MSS)**: This method calculates the cluster weight similarly to **MS** except that it does not average the node similarity: $w_{ij}=\overset{\left|c_{i}\right|}{\underset{k=1}{\sum }}\overset{\left|c_{j}\right|}{\underset{l=1}{\sum }}r_{kl}^{ij}\cdot sim\left(v_{k},v_{l}\right)$.

###### 6.1.2.2.3. Cluster graph

Once the cluster similarities have been determined, we need to decide what similarity constitutes a potential cluster match. Such potential matches are added as edges in the cluster graph which is considered for maximum weighted matching. We consider three different options:

- **Threshold-based cluster matching (TCM)**: Cluster *c*_*i*_ is considered as a potential match of cluster *c*_*j*_ if their weight *w*_*i, j*_ > *β*. The threshold *β* can vary from 0, which allows all pairs of clusters to be potential matches, to the maximum possible similarity which allows matching between clusters only if they are exactly the same. In our experiments, we set *β* based on the distribution of pairwise similarities and their quartiles as thresholds.
- **Greedy cluster matching (GCM)**: For each cluster *c*_*i*_, a sorted list of the similarities between *c*_*i*_ and all other clusters is defined. Cluster *c*_*i*_ is considered a potential match only to the cluster with the highest similarity value in the list.

The last step in *CMatch* runs maximum weighted matching on the cluster graph. For every matched cluster pair, it assigns one cluster to treatment and the other one to control at random. This completes the network experiment design.

#### 6.1.3. Analysis of the estimation bias

We follow Eckles et al. (2016) to analyze the estimation bias of the proposed cluster-based approach. One of the common approaches to measuring the causal effect *μ* of a treatment on an outcome is averaging outcomes over treatment and control groups *via* difference-in-means: $\mu^{d}=\mu^{d}(V_{1}.Y)-\mu^{d}(V_{0}.Y)$ where $\mu^{d}\left({\text{V}}_{1}.Y\right)$ and $\mu^{d}\left({\text{V}}_{0}.Y\right)$ are the mean outcomes of treatment and control nodes under experiment design d, respectively. In the presence of interference, μ^*d*^ does not yield the true total treatment effects (*μ*^*d*^ − *μ* ≠ 0). The impact of each node on the estimation bias is equal to the difference between the expected outcome of a node due to the treatment alone and the observed outcome under global treatment assignment where all nodes in the network have a treatment assignment. The experimental design can control the size of this bias by controlling the global treatment assignment. Eckles et al. prove that this bias in the cluster-based randomization approach is less than or equal to the absolute bias under randomized assignment. Following this study, if we assume that we have a linear outcome model for each node *v*_*i*_ ∈ **V** as Eckles et al. (2016):


(10) $$\begin{array}{l} E\left[v_{i}.Y\left(\text{Z}\right)\right]=a_{i}+\underset{v_{j}\in V}{\sum }B_{ij}v_{j}.T, \end{array}$$

Where **B** is the coefficient matrix, then true TTE *μ* is calculated as Eckles et al. (2016):


(11) $$\mu =\mu (Z_{1})-\mu (Z_{0})=\frac{1}{n}\sum_{ij}B_{ij}.$$

Under cluster-based randomization assignment, we have Eckles et al. (2016):


(12) $$\begin{array}{l} \mu^{cbr}=\frac{1}{n}\underset{v_{i},v_{j}\in V}{\sum }B_{ij}1\left[C\left(v_{i}\right)=C\left(v_{j}\right)\right], \end{array}$$

Where *C*(*v*_*i*_) denotes the cluster assignment of *v*_*i*_. Under randomized assignment, we have Eckles et al. (2016):


(13) $$\begin{array}{l} \mu^{rand}=\frac{1}{n}\underset{v_{i}\in V}{\sum }B_{ii}. \end{array}$$

Equations (11)–(13) imply that *μ* − *μ*^*cbr*^ ≤ *μ* − *μ*^*rand*^. The effectiveness of cluster-based randomization in reducing bias depends on the strength of interactions within clusters. The ability of the clustering algorithm to capture the coefficient matrix **B** in a consistent manner also affects the degree of bias reduction. By incorporating the strength of connections between units into the clustering process, the method can better capture the structure of dependence between units, resulting in a smaller bias (*μ* − *μ*^*cbr*^). Considering Equations (11)–(12), the relative bias is measured as Eckles et al. (2016):


(14) $$\frac{\mu }{\mu^{cbr}}-1=\frac{\sum_{v_{i},v_{j}\in V}B_{ij}1[C(v_{i})=C(v_{j})]}{\sum_{v_{i},v_{j}\in V}B_{ij}}-1.$$

If the clustering fails to capture the structural dependencies, the numerator in Equation (14) will be much smaller than the denominator. As a result, the method will underestimate the true total treatment effects.

## 7. Experiments

In this section, we evaluate the performance of *CauseIS* and *CMatch* in treatment effect estimation compared to the baselines. We first describe datasets used in our experiments and then discuss the experimental setup and results.

### 7.1. Data generation

Since existing network datasets do not have ground truth for treatment and its causal effect on the outcome, we use synthetic and real-world data structures and simulate the outcome and causal effect in the experiments.

#### 7.1.1. Synthetic data

For generating synthetic networks, we use two network generation models:

- *Barab*á*si-Albert (BA)* model: This model generates random scale-free networks using the preferential attachment model. In the beginning, the network is constructed from *m*_0_ connected nodes. Then, new nodes are connected to *m* existing nodes with a probability that is proportional to the number of edges that the existing nodes already have (Albert and Barabási, 2002). We set *m* = 3 in all experiments.
- *Forest Fire (FF)* model: In this model, a new node *v*_*i*_ attaches to an existing node *v*_*j*_ and then links to nodes connected to *v*_*j*_ with forward and backward burning probabilities denoted by *p*_*f*_ and *p*_*b*_, respectively. Leskovec et al. (2007) show that the synthetic network generated by this model can mimic most real-world structure characteristics. In the experiments, we generate all the graphs with forward burning probability *p*_*f*_ = 0.3 and backward burning probability *p*_*b*_ = 0.3.

After generating the network structure, we generate 10 attributes for each node with a uniform distribution where the values vary in [ − 1, 1].

#### 7.1.2. Real-world data

We use five real-world datasets in our experiments. The *50 Women* dataset (Michell and Amos, 1997) includes sport, smoking, drug, and alcohol habits of 50 students with 74 friendship connections. *Cora* and *Citeseer* datasets (Sen et al., 2008) incorporate the citation networks of 2, 708 and 3, 312 article with binary bag-of-words attributes for each article and 4, 675 and 5278 edges, respectively. *Hamsterster* dataset (Zheleva et al., 2008) includes the online friendship network of 2, 059 hamsters with 10, 943 edges. *Hateful users* dataset (Ribeiro et al., 2018) is a sample of Twitter's retweet graph containing 100, 386 users with 1, 024 attributes and more than two millions retweet edges. In *hateful users* dataset, we remove singletons and nodes with degree 1 from the graph.

#### 7.1.3. Synthetic causal effect

We assume that the underlying probability of activating a node (changing the outcome) due to treatment and allowable peer effects in the treatment group is 0.4 and the underlying probability of activating a control node due to treatment and allowable peer effects is 0.2 which makes the true causal effect *TTE* = 0.2. Based on these probabilities, we randomly assign each node as activated or not. For each inactivated node, we simulate two types of interference considering both fixed values (0.1 and 0.5) and values based on the edge weights for *e*.*p*:

1. Direct interference: each treated neighbor of a control node activates the node with an unallowable spillover probability of *e*.*p*.
2. Contagion: inactive treated and untreated nodes get activated with the unallowable spillover probability of *e*.*p* if they are connected to at least one activated node in a different treatment class.

### 7.2. Main algorithms and baselines

Our baselines differ corresponding to the causal effect of interest. In the following, we describe the main baselines for direct and total treatment effect estimation.

#### 7.2.1. Baselines for Direct Treatment Effect Estimation

We compare the performance of four different approaches in our experiments.

- **Randomized**: This algorithm assigns nodes to treatment and control randomly, ignoring the network.
- **Match**: This algorithm matches nodes using the maximum weighted matching algorithm and then randomly assigns nodes in each matched pair to treatment and control at random without considering clustering.
- **CauseIS**: In our proposed framework, we use an algorithm to find the maximum independent set *MIS* and then assign nodes of the set to treatment or control at random.
- **CauseIS_match**: This method uses the *CauseIS* framework, but it matches nodes of *MIS* and then assigns nodes of matched pairs to treatment or control at random.

The goal of comparing our method with *Match* and *CauseIS*_*Match* is to show whether our method has selection bias. Using matching for RCT is unusual, but in small datasets altering the randomization process by posing structural constraints on the graph may lead to worse randomization and matching can mitigate this problem.

#### 7.2.2. Baselines for total treatment effect estimation

For TTE, all our baseline and main algorithm variants take an attributed graph as an input and produce a set of clusters, each assigned to treatment, control, or none. For graph clustering, we considered two main algorithms, *Restreaming Linear Deterministic Greedy (reLDG)* (Nishimura and Ugander, 2013) and *Markov Clustering Algorithm (MCL)* (Enright et al., 2002). *reLDG* takes as input an unweighted graph and desired the number of clusters and produces a graph clustering. *reLDG* was reported to perform very well in state-of-the-art methods for network experiment design (Saveski et al., 2017). *MCL* is a non-parametric algorithm that takes as input a weighted graph and produces a graph clustering. The edge weights which correspond to the probabilities of spillover are estimated based on node pair similarity using one minus the normalized L2 norm: 1 − *L*_2_(*v*_*i*_.*x, v*_*j*_.*x*).

The main algorithms and baselines are:

- **CR** (Saveski et al., 2017): The *Completely Randomized (CR)* algorithm was used as a baseline in Saveski et al. (2017). The algorithm clusters the unweighted graph using *reLDG* algorithm, assigns similar clusters to the same strata, and assigns nodes in strata to treatment and control in a randomized fashion.
- **CBR_reLDG_** (Saveski et al., 2017): *Cluster-based Randomized assignment (CBR)* is the main algorithm proposed by Saveski et al. (2017). The algorithm clusters the unweighted graph using *reLDG*, assigns similar clusters to the same strata, and randomly picks clusters within the same strata as treatment or control.
- **CBR_MCL_**: A variant of **CBR** that we introduce for the sake of fairness which uses *MCL* for weighted-graph clustering.
- **CMatch_reLDG_**: This method uses our *CMatch* framework but works on an unweighted graph. It uses *reLDG* for graph clustering.
- **CMatch_MCL_**: This is our proposed technique which uses *MCL* for weighted graph clustering.

We consider *Randomized* and *Match* techniques described in Section 7.2.1 as two more baselines for total treatment effect estimation. *CMatch* uses the *maximum*_*weight*_*matching* function from the *NetworkX* Python library.

### 7.3. Experimental setup

We run a number of experiments varying the underlying spillover assumptions, clustering algorithms, number of clusters, and node matching algorithms. Our experimental setup measures the desired properties for network experiment design, as described in Problem 2 and follows the experimental setups in existing work (Stuart, 2010; Maier et al., 2013; Arbour et al., 2014; Eckles et al., 2016; Saveski et al., 2017).

To measure the strength of interference bias in different estimators, we report on two metrics:

1. *Root Mean Squared Error* (RMSE) of the treatment effect calculated as:
  $$\begin{array}{l} RMSE=\sqrt{\frac{1}{S}\overset{S}{\underset{s=1}{\sum }}\left({\left({\hat{\tau }}_{s}-\tau_{s}\right)}^{2}\right)} \end{array}$$
  where *S* is the number of runs and τ_*s*_ and ${\hat{\tau }}_{s}$ are the true and estimated causal effect in run *s*, respectively. We set *S* = 10 in all experiments. The error can be attributed to undesired spillover only.
2. The number of edges and the sum of edge weights between treatment and control nodes are assigned by each algorithm.

To show the selection bias, we want to assess how different treatment vs. control nodes are. We compute the Euclidean distance between the attribute vector mean of treated and untreated nodes. We show the average and standard deviation over 10 runs.

To show the strength of UPE imposed by bystander nodes in the *CauseIS* framework, we calculate the difference between the percentage of edges from bystander nodes to treatment and control nodes as:


(15) $$\begin{array}{l} \frac{1}{\left|\text{E}\right|}\left(\underset{\begin{array}{c} e_{i,j}\in \text{E}\\ v_{i}\in \text{T}\\ v_{j}\in \text{B} \end{array}}{\sum }d_{i,j}-\underset{\begin{array}{c} e_{i,j}\in \text{E}\\ v_{i}\in \text{C}\\ v_{j}\in \text{B} \end{array}}{\sum }d_{i,j}\right)\times 100 \end{array}$$

Where *d*_*i,j*_ = 1 if there is an edge between node *v*_*i*_ and *v*_*j*_. *T* and *C* show the vector of treatment and control nodes.

in our experiments, we use the *maximal*_*independent*_*set* function from the *NetworkX* Python library to find a maximal independent set of each graph which implements the approach by Blelloch et al. (2012).

We run all 115 possible combinations of CMatch options for node matching, cluster weights, and cluster graphs for each dataset. We consider four different values for the threshold *α* in **TNM**: 0 (**TNM0**), first (**TNM1**), second (**TNM2**) and third (**TNM3**) quantile of pairwise nodes' similarity distribution where *sim*(*v*_*i*_, *v*_*j*_)= (1- the normalized *L*_2_ norm). For **TCM**, we consider four different *β* values: 0 (**TCM0**), first (**TCM1**), second (**TCM2**) and third (**TCM3**) quantile of the pairwise clusters' similarity distribution for each dataset. We use **TNM2 + C + TCM2** in all the experiments of *CMatch*_*reLDG*_.

Unless otherwise specified, the number of clusters is the same for all CBR and CMatch versions based on the optimal determination by MCL as optimal for each respective dataset. The number of clusters determined by *MCL* is 2, 497 for *Citeseer*, 1, 885 for *Cora*, 1, 056 for *Hamsterster* and 20 in *50 Women* dataset.

### 7.4. Results

Here, we present the experimental results for the proposed framework. We first describe the performance of the CauseIS approach in estimating direct treatment effects. Then, we show the effectiveness of the CMatch framework in mitigating interference and selection bias.

#### 7.4.1. Performance of *CauseIS* framework

##### 7.4.1.1. Evaluation of direct treatment effect estimation

**Figure 4:**
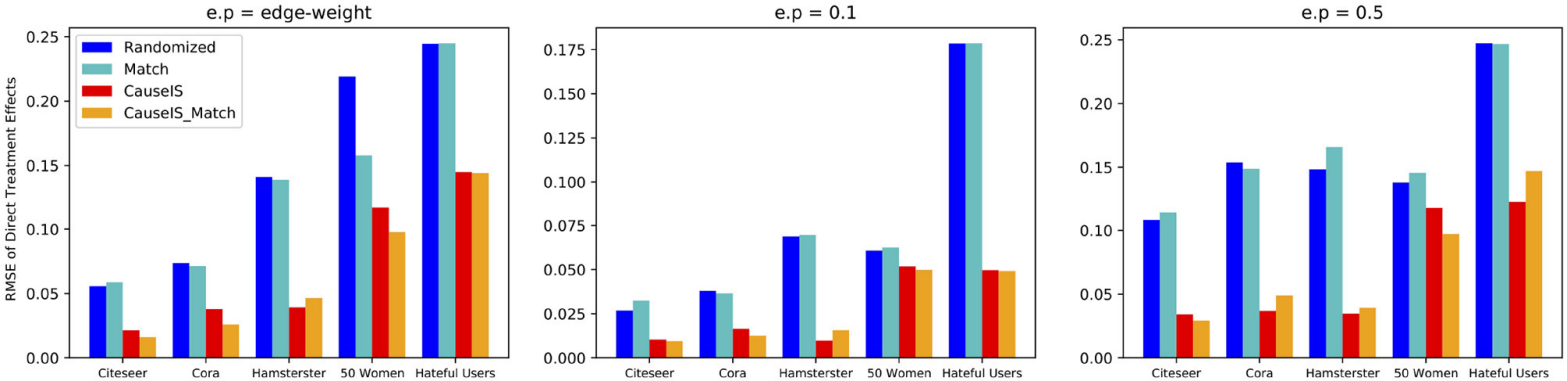
RMSE of direct treatment effects in real-world datasets considering different unallowable peer effect probabilities.

To assess the accuracy of *CauseIS* in estimating DTE compared to the baselines, we measure causal effect estimation error for different unallowable peer effect probabilities. Figure 4 shows the RMSE of DTE in real-world data sets. In all five datasets, *CauseIS* and *CauseIS*_*Match* get lower estimation error, compared to *Randomized* and *Match*, especially in *Hamsterster* with 72.1% and 76.6% estimated error reduction for *e*.*p* = *edge*_*weight* and *e*.*p* = 0.5 and *Hateful Users* with 69.4% estimated error reduction for *e*.*p* = 0.1. By increasing the spillover probability from 0.1 to 0.5, we get higher estimation errors because the probability of changing treatment and control outcomes through peer effects increases.

**Figure 5:**
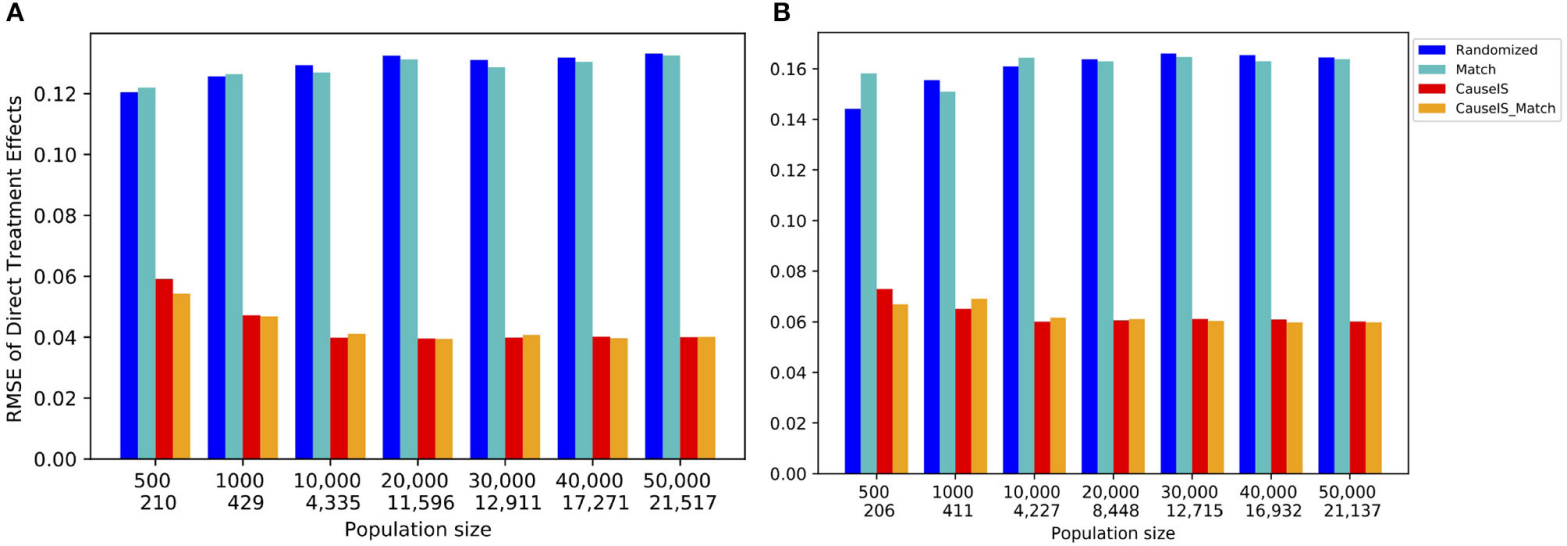
RMSE of direct treatment effect in synthetic data with a different number of nodes and edges. Numbers in the first row of the x-axis show the number of nodes in graphs, and the second row represents the size of MIS. **(A)** Forest Fire model. **(B)** Barab'asi-Albert model.

Synthetic data experiments depict a similar picture. Figure 5 shows the stronger performance of *CauseIS* and *CauseIS*_*Match* over *Randomized* and *Match* methods in reducing causal effect estimation error. For example, *CauseIS*'s error is more than half of the error of *Randomized* approach (0.04 vs. 0.12 for graphs with 10, 000 nodes, 0.13 vs. 0.035 for graphs with 20, 000 nodes in Forest Fire model). In graphs with 50, 000 nodes, *CauseIS* obtains 63.4% and 69.9% estimation error reduction in Forest Fire and Barabási-Albert models respectively, compared to other graphs.

In both synthetic and real-world datasets, *Randomized* and *Match* on one hand and *CauseIS* and *CauseIS*_*Match* on the other hand show similar performances. This is intuitive because they use similar randomization techniques. While MIS size is approximately half of the population size in all datasets, by increasing the size of MIS the estimation error of *CauseIS* is still significantly lower than *Randomized* methods with smaller population size.

##### 7.4.1.2. Sensitivity to the density of networks

**Figure 6:**
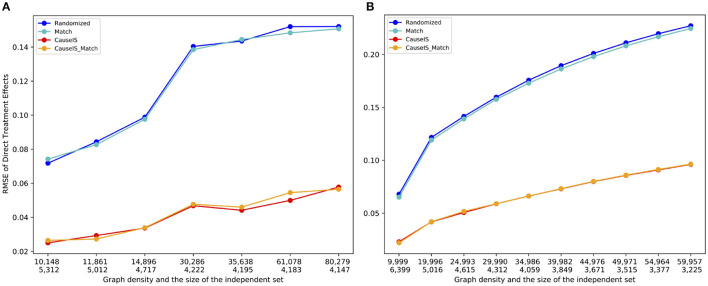
RMSE of direct treatment effects in synthetic data with 10, 000 nodes and different densities. Numbers in the first row of the x-axis show the number of edges in graphs, and the second row represents the size of MIS. **(A)** Forest Fire model. **(B)** Barab'asi-Albert model.

To assess the impact of network density on the estimation error of various models, we computed the average estimation error across 10 randomly generated graphs containing 10,000 nodes for each density value. We adjusted the density of graphs in the Barabási-Albert model by altering the value of *m* within the range of 1–9, while for the Forest Fire model, we set *p*_*f*_ = *p*_*b*_ and varied *p*_*f*_ between 0.01 and 0.35. Figure 6 illustrates that as the density of the graphs increases, the estimation error for all methods also increases. This observation is expected since an increase in the number of edges between treatment and control raises the possibility of unallowable peer effects in the experiment. However, the *CauseIS* and *CauseIS*_*Match* methods consistently outperform the other two baseline methods in all graphs. Moreover, an increase in the density of the graph leads to a decrease in the size of the MIS. A higher MIS rate (meaning fewer bystander nodes) implies fewer spillover effects from bystander nodes to treatment and control, resulting in smaller estimation errors.

##### 7.4.1.3. Selection bias evaluation

**Figure 7:**
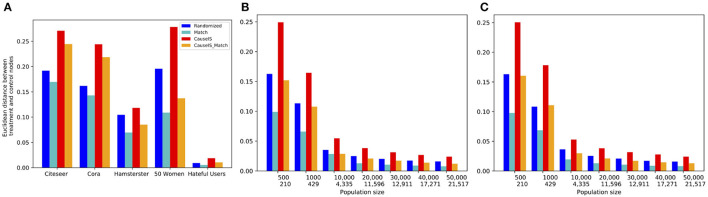
Euclidean distance between the attribute vector means of treatment and control nodes in real-world and synthetic datasets. In synthetic dataset plots, numbers in the first row of the x-axis show the number of nodes in graphs, and in the second row show the size of MIS. **(A)** Real-world data, **(B)** Forest Fire model, and **(C)** Barab'asi-Albert model.

In this experiment, we evaluate the selection bias of different methods by comparing the Euclidean distance between treatment and control nodes' attributes in real-world and synthetic datasets with different population sizes. Figure 7 shows this comparison of real-world and synthetic data. It is not surprising that the *Match* method gets the lowest selection bias in all datasets because it matches most similar treatment and control nodes based on the similarity of attributes. *CauseIS*_*Match* has a higher selection bias than *Match* because the number of nodes matched in this approach is less than the *Match* method. Although *CauseIS* has a high selection bias, *CauseIS*_*Match* reduces selection bias to some extent.

Next, we look at how sample size impacts selection bias. We expect that asymptotically, there would be no selection bias with randomization for any design. Figure 7 shows that independent from the network generating model, by increasing the population size the similarity between treatment and control nodes' attributes reduces, and the value of matching decreases and disappears. For example, in graphs with 500 nodes generated by the Forest Fire model, the difference between Euclidean distance of treatment and control nodes in *CauseIS* is 0.24, while in graphs with 50, 000 nodes, this difference decreases to 0.024. These results confirm the advantage of the matching technique in small datasets.

##### 7.4.1.4. Peer effect evaluation

**Figure 8:**
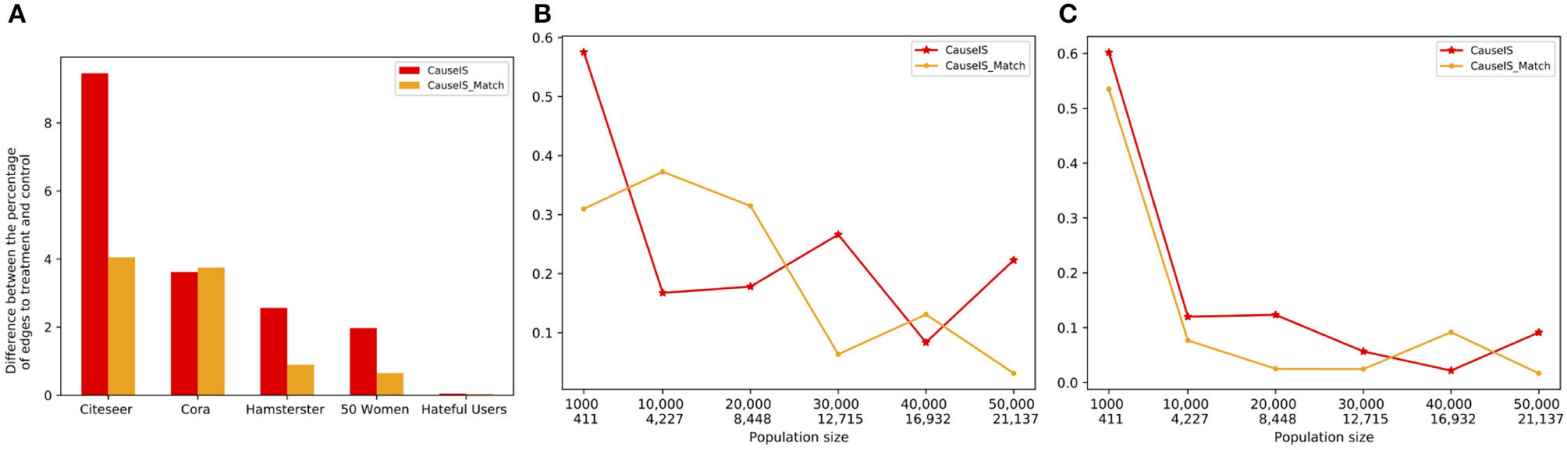
Difference between the percentage of edges to treatment and control nodes in real-world and synthetic datasets with a different number of nodes and edges. In synthetic dataset plots, numbers in the first row of the x-axis show the number of nodes in graphs, and in the second row show the size of MIS. **(A)** Real-world data, **(B)** Forest Fire model, and **(C)** Barab'asi-Albert model.

To measure the extent to which UPE(**V**_0_) and UPE(**V**_1_) can cancel each other out, we consider the percentage of edges from bystander nodes to treatment and control nodes. Figure 8 shows this quantity in real-world and synthetic datasets using *CauseIS* and *CauseIS*_*Match* methods. As expected, results show that for graphs with fewer number of nodes, the difference between the number of edges to treatment and control nodes is higher compared to larger graphs, 2.5 vs. 0.04 in *50 Women* vs. *Hateful Users* dataset. In synthetic data with higher population sizes (40, 000 and 50, 000), the difference between the percentages of edges to treatment and control is close to zero.

In both synthetic and real-world datasets, we observe that by increasing the sample size, the causal effect estimation error decreases because by increasing the density of the graph edges the percentage of edges from bystander nodes to treatment and control nodes becomes more similar and UPE(**V_1_**) - UPE(**V_0_**) goes to zero.

##### 7.4.1.5. Degree distribution evaluation

**Figure 9:**
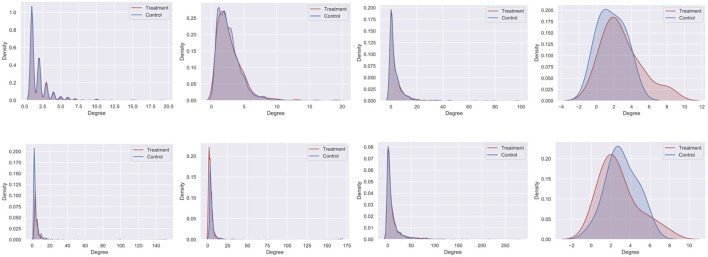
Degree distribution of treatment and control nodes selected by *CauseIS* **(first row)** and *Randomized* **(second row**).

To assess the extent to which the maximal independent set chosen by *CauseIS* biases the degree distribution of selected treatment and control nodes, we compare the degree distributions of treatment and control nodes selected by *CauseIS* and *Randomized*. Figure 9 shows that *CauseIS* selects treatment and control groups with roughly similar degree distribution in all datasets, except in *50 Women* dataset where the assignment looks more biased, likely due to its small size. *CauseIS* removes high-degree nodes from the experiment which results in incorporating treatment and control groups with a more balanced degree distribution in the experiments.

#### 7.4.2. Performance of *CMatch* framework

##### 7.4.2.1. Tradeoff between interference and selection bias in *CMatch* variants and baselines

Given the large number of *CMatch* option combinations (115), we first find which ones of these combinations have a good tradeoff between RMSE and Euclidean distance (between treatment and control) with *e.p = edge-weight*. Depending on the node matching and cluster matching thresholds, which are specified by the user, the performance of *CMatch* options varies. Based on these experiments, we notice that 1) methods with stricter cluster thresholds (**TCM2** and **TCM3**) tend to have a lower error, 2) stricter node match thresholds (**TNM2** and **TNM3**) have lower error than others for **S** and **MSS** and 3) **MS** has high error across thresholds. We show the detailed results for Cora in Table 1.

**Table 1:** The tradeoff between selection bias (distance) and undesirable spillover (RMSE) in *CMatch* variants in the Cora dataset.

		**TCM0**		**TCM1**		**TCM2**		**TCM3**		**GCM**	
		**RMSE**	**ED**	**RMSE**	**ED**	**RMSE**	**ED**	**RMSE**	**ED**	**RMSE**	**ED**
C	TNM0	0.052	0.184	0.007	0.267	0.017	0.263	0.014	0.26	0.048	0.789
	TNM1	0.055	0.176	0.051	0.177	0.008	0.258	0.012	0.26	0.031	0.6
	TNM2	0.054	0.171	**0.042**	**0.171**	**0.01**	**0.253**	0.017	0.251	0.036	0.591
	TNM3	0.043	0.175	0.043	0.175	0.0173	0.046	0.018	0.231	0.034	0.592
	BNM	0.012	0.262	0.037	0.481	0.049	0.485	0.059	0.479	0.025	0.274
S	TNM0	0.056	0.16	0.058	0.159	0.048	0.16	0.056	0.162	0.035	0.34
	TNM1	0.055	0.16	0.053	0.162	0.057	0.165	0.054	0.166	0.026	0.31
	TNM2	0.056	0.162	0.054	0.168	0.048	0.165	0.033	0.183	0.039	0.292
	TNM3	0.057	0.169	0.041	0.174	0.024	0.198	**0.015**	**0.211**	0.021	0.275
	BNM	0.014	0.253	0.017	0.264	0.02	0.27	0.027	0.303	0.014	0.277
MC	TNM0	0.049	0.177	0.015	0.261	0.01	0.262	0.008	0.263	0.042	0.189
	TNM1	0.055	0.173	0.052	0.174	0.01	0.257	0.012	0.253	0.040	0.191
	TNM2	0.047	0.171	0.051	0.177	0.013	0.261	0.007	0.263	0.024	0.211
	TNM3	0.047	0.173	0.049	0.178	0.051	0.176	0.011	0.249	0.012	0.244
	BNM	N/A	N/A	N/A	N/A	N/A	N/A	N/A	N/A	N/A	N/A
MS	TNM0	**0.048**	**0.155**	0.051	0.156	0.052	0.156	0.058	0.157	0.018	0.271
	TNM1	0.051	0.156	0.057	0.157	0.048	0.156	0.052	0.16	0.022	0.264
	TNM2	0.059	0.156	0.057	0.157	0.054	0.158	0.056	0.157	0.021	0.258
	TNM3	0.053	0.157	0.05	0.159	0.056	0.155	0.051	0.156	0.028	0.27
	BNM	N/A	N/A	N/A	N/A	N/A	N/A	N/A	N/A	N/A	N/A
MSS	TNM0	0.059	0.162	0.048	0.162	0.061	0.159	0.036	0.184	0.026	0.271
	TNM1	0.056	0.16	0.054	0.161	0.047	0.161	0.03	0.194	0.029	0.275
	TNM2	0.052	0.161	0.057	0.161	0.045	0.172	**0.028**	**0.195**	0.021	0.281
	TNM3	0.049	0.168	0.035	0.186	0.023	0.199	0.022	0.212	0.033	0.278
	BNM	N/A	N/A	N/A	N/A	N/A	N/A	N/A	N/A	N/A	N/A
E	N/A	0.051	0.178	0.05	0.18	0.031	0.203	0.012	0.242	0.042	0.718

**Figure 10:**
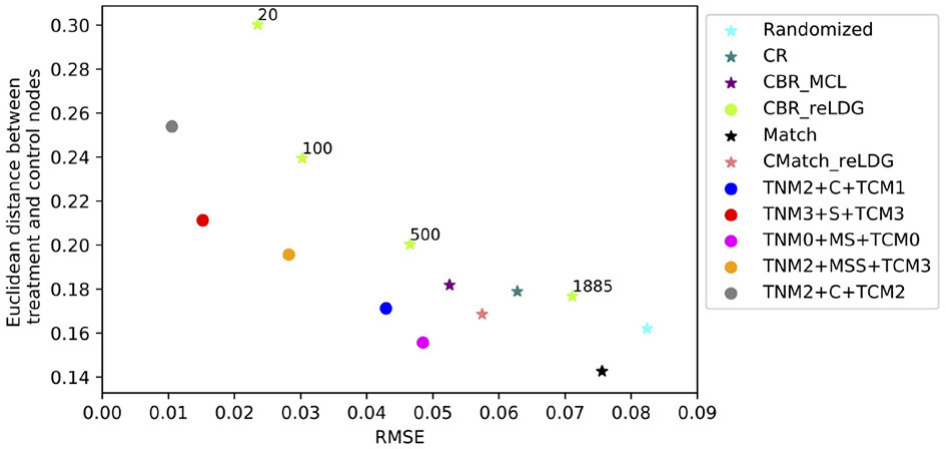
The tradeoff between selection bias (distance) and undesirable spillover (RMSE) in *CMatch*_*MCL*_ variants (labeled with methods applied in) and baselines in the Cora dataset for *e.p = edge-weight*; *CBR*_*reLDG*_ is annotated with the number of clusters.

*CMatch*_*MCL*_ variants used in Figure 10 are in bold.

Figure 10 shows the results for the *CMatch* variants with the best tradeoffs and their better performance when compared to the baselines for Cora. Full *CMatch* results can be found in Table 1. The figure clearly shows that the selection bias decreases at the expense of interference bias. For example, while the Euclidean distance for **TNM0 + MS + TCM0** is low (0.155) when compared to **TNM2 + C + TCM2** (0.253), its RMSE is higher, 0.048 vs. 0.01. The comparison between *CBR*_*reLDG*_ with different possible number of clusters is consistent with the tradeoff shown in Figure 1. *CBR*_*reLDG*_ with the highest error (annotated with 1885) and *CMatch*_*MCL*_ have the same number of clusters. It is intuitive that the *Match* method has the least selection bias because all nodes have their best matches. However, similar to the *Randomized* method, it suffers from high interference bias (RMSE) because of the high density of edges between treatment and control nodes.

##### 7.4.2.2. Interference evaluation for contagion

**Figure 11:**
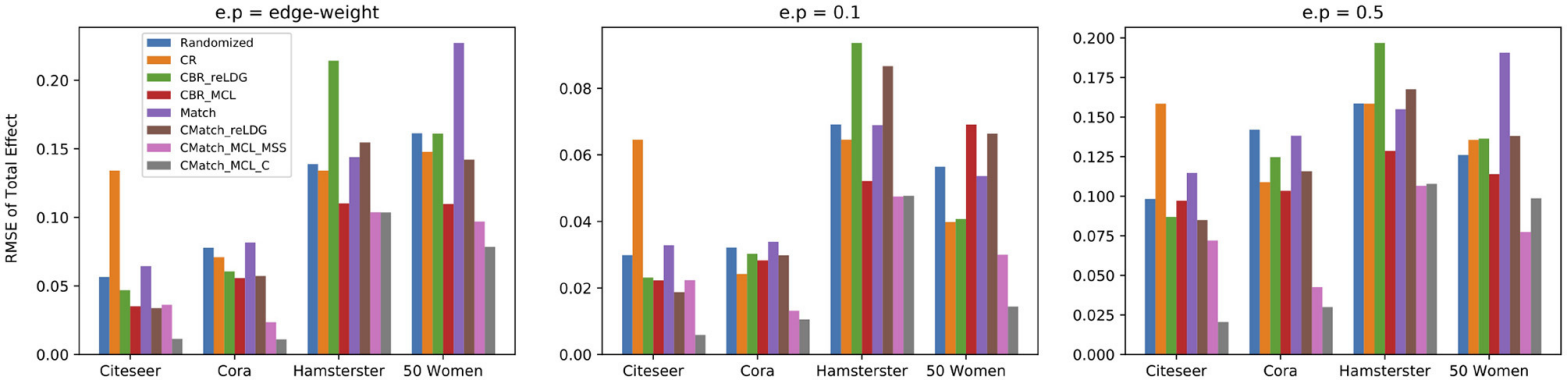
RMSE of total effect in the presence of contagion considering different unallowable spillover probabilities in all datasets; *CMatch*_*MCL*_*C*__ achieves the lowest error in all datasets.

We choose two *CMatch* variants with low estimation errors: **TNM2 + MSS + TCM3** and **TNM2 + C + TCM2**, denoted by *CMatch*_*MCL*_*MSS*__ and *CMatch*_*MCL*_*C*__ respectively, and compare their causal effect estimation error with the baselines. The first method uses a simpler cluster weight assignment while the second one uses the expensive maximum weighted matching of nodes. Figure 11 shows that both variants of *CMatch*_*MCL*_ get significantly lower error than other methods, especially in Citeseer and Cora with 75.5% and 81.8% estimated error reduction in comparison to *CBR*_*reLDG*_ for *e.p = edge-weight*. *CMatch*_*MCL*_*MSS*__ has higher error than *CMatch*_*MCL*_*C*__ in most of the experiments which are expected as shown in Figure 10. *Randomized* and *Match* approaches have similar performance in all datasets because of their similarity in the node randomization approach. We also notice that *CBR*_*reLDG*_ has the highest estimation error in Hamsterster data which confirms that clustering has a significant effect on the unallowable spillover. Meanwhile, *CMatch*_*reLDG*_ outperforms other baselines in some datasets (Citeseer) but not in others (Hamsterster and 50 Women). In Citeseer, the *CR* method gets the largest estimation error.

Figure 11 also shows that the higher the unallowable spillover probability, the larger the estimation error but also the better our method becomes relative to the baselines. For example, by increasing the unallowable spillover probability from 0.1 to 0.5 in Citeseer, the estimation error increases from 0.005 to 0.02 for *CMatch*_*MCL*_*C*__ and from 0.023 to 0.086 for *CBR*_*reLDG*_.

##### 7.4.2.3. Interference evaluation for direct interference

**Figure 12:**
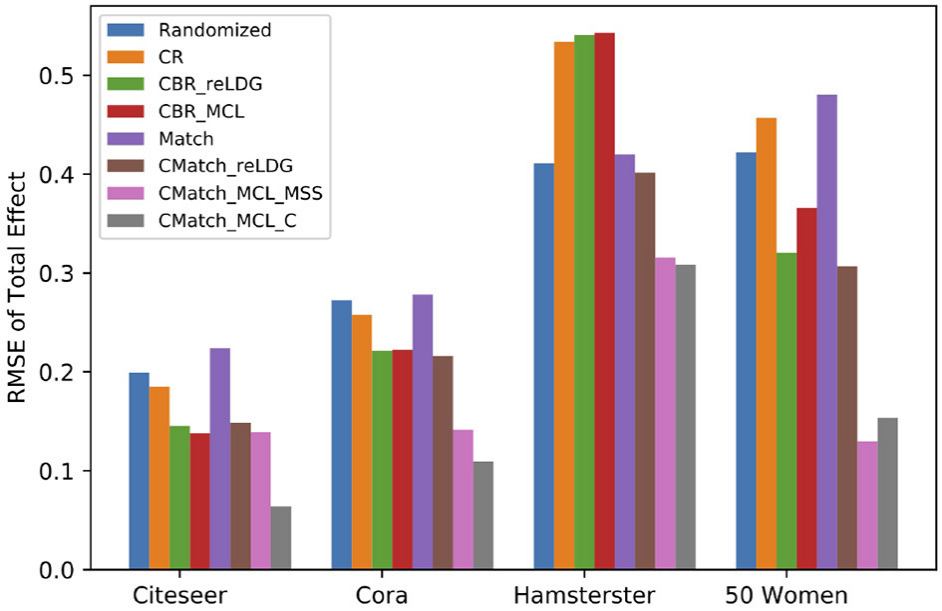
RMSE of total effect in the presence of direct interference (*e.p = edge-weight*). *CMatch*_*MCL*_*C*__ and *CMatch*_*MCL*_*MSS*__ obtain the lowest RMSE for all datasets.

Figure 12 shows the difference between the RMSE of different estimators over the presence of direct interference for *e.p = edge-weight*. In four datasets, both variants of *CMatch*_*MCL*_ get the lowest estimation error in comparison to baseline methods. For example, *CMatch*_*MCL*_*C*__'s error is approximately half of the error of *CBR*_*reLDG*_ (0.06 vs. 0.13 for Citeseer, 0.1 vs. 0.22 for Cora, 0.31 vs. 0.54 for Hamsterster, 0.15 vs. 0.36 for 50 Women). Similar to contagion, *Match*, and *Randomized* methods have similar estimation errors.

##### 7.4.2.4. Potential spillover evaluation

Table 2 shows the potential spillover between treatment and control nodes assigned by different methods. This applies to both contagion and direct interference. *CMatch* has the lowest sum of edges and edge weights between treatment and control nodes across all datasets. The difference between *CMatch*_*MCL*_*C*__ and the baselines in Cora and Citeseer is substantial: *CMatch*_*MCL*_*C*__ has between 13.5 and 34.8% lower number of edges between treatment and control across datasets.

**Table 2:** Percentage of edges (and edge weights) between treatment and control nodes.

**Dataset**	**Randomized**	**CR**	***CBR*_*reLDG*_**	***CBR*_*MCL*_**	**Match**	***CMatch*_*reLDG*_**	***CMatch*_*MCL*_*C*__**
Citeseer	49.9% (50%)	35.9% (36.3%)	39.8% (38.4%)	38.9% (38.4%)	53.9% (56.6%)	35.8% (34.4%)	**7.5%** (7.2%)
Cora	49.7% (49.7%)	37.6% (37.6%)	43.4% (42.8%)	38.9% (33.6%)	51.8% (53.3%)	38.7% (38.2%)	**8.6**% (9.1%)
Hamsterster	50.2% (50.1%)	31.7% (30.4%)	48.3% (48.3%)	35.1% (34.7%)	50% (50.1%)	43.3% (44.4%)	**34.8%** (34.4%)
50 Women	48.5% (48.1%)	31.8% (30.5%)	36.6% (34.3%)	18.3% (11.4%)	52.5% (52.7%)	16% (18.6%)	**12.8%** (9.7%)

The lower the number, the lower probability of undesired spillover. The smallest percentages are shown in bold.

##### 7.4.2.5. Selection bias evaluation for contagion

**Figure 13:**
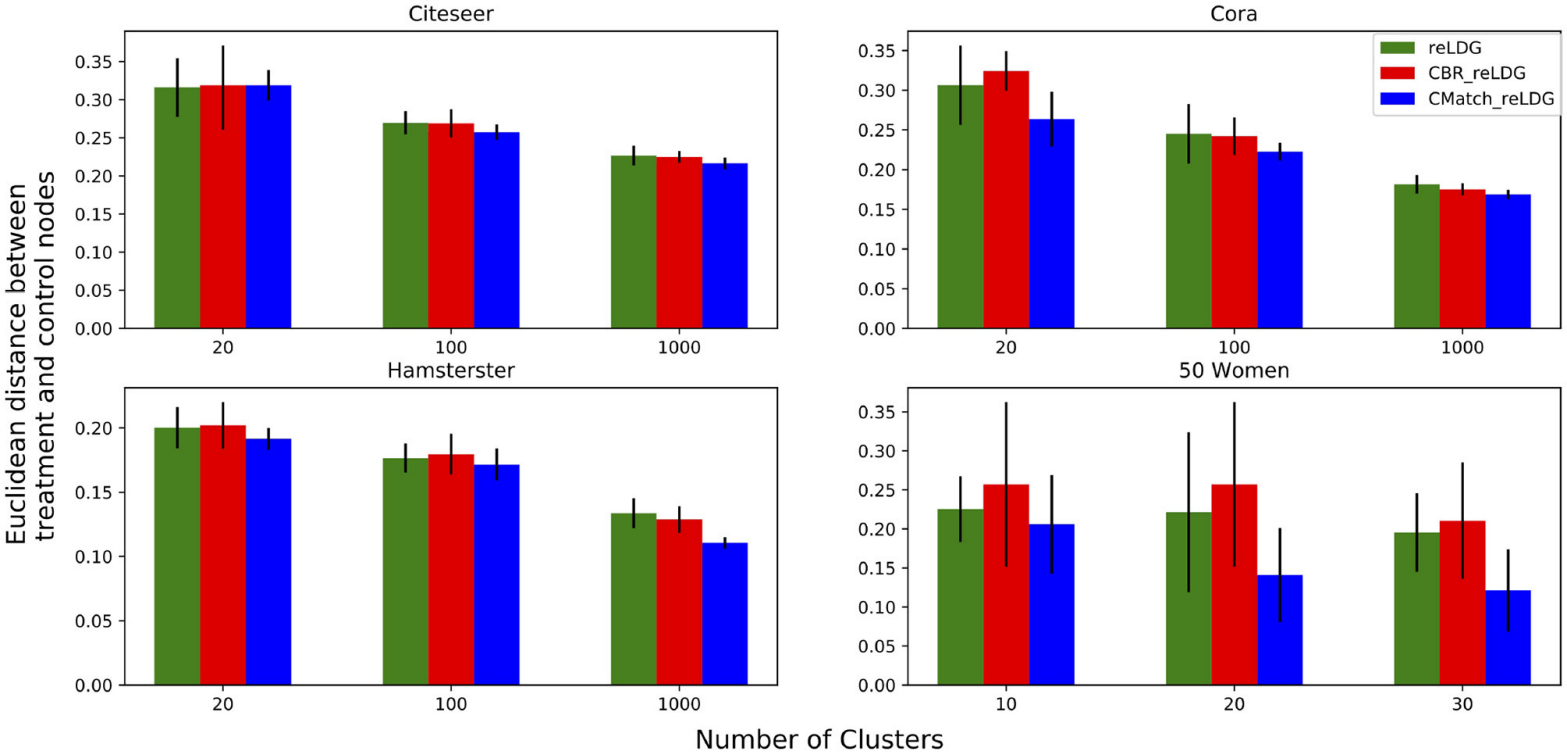
Euclidean distance between the attribute vector means of treatment and control nodes for a different number of clusters. The higher the number of clusters, the lower the selection bias.

In this experiment, we look at the relationship between the number of clusters and the difference between treatment and control nodes with and without cluster matching. Figure 13 shows the Euclidean distance between the average of treatment and control nodes' attributes in *CMatch*_*reLDG*_, *CBR*_*reLDG*_ and *reLDG* for three different numbers of clusters and unallowable spillover probability *e.p = edge-weight*. Since *CMatch*_*reLDG*_ optimizes for selection bias directly, it is not surprising that it results in treatment and control nodes that have more similar feature distributions than the other two methods. In Citeseer the differences are more subtle than in the other datasets. Error bars show the variance of averages over 10 runs which confirm the low variance of estimations in all datasets except in 50 Women, which is a small dataset.

##### 7.4.2.6. Sensitivity to spillover probability metrics

**Figure 14:**
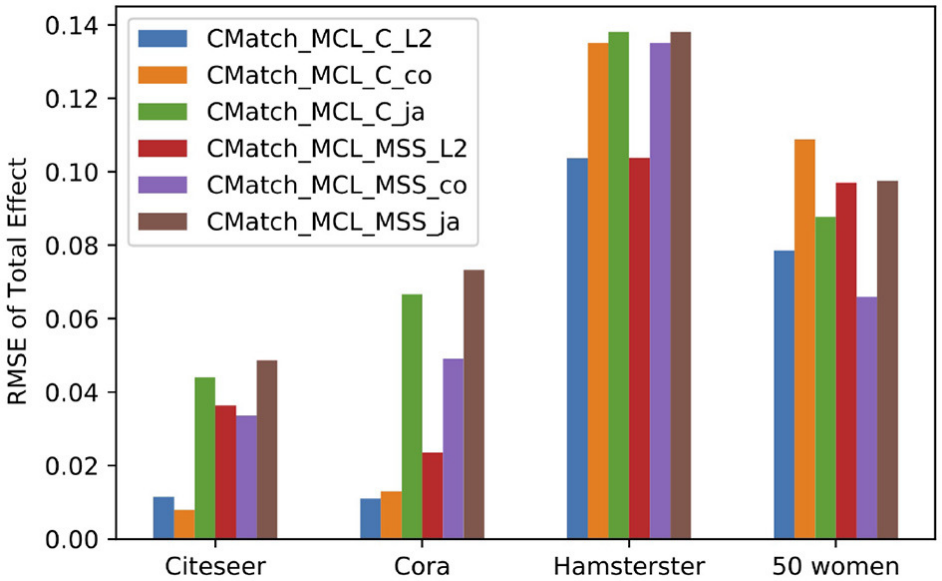
RMSE of total effect in the presence of contagion using three different similarity methods to calculate spillover probability: Cosine (co), Jaccard (ja) and L2 similarity.

Our last experiment compares metrics for calculating the spillover probability, Cosine similarity, Jaccard similarity, and the L2-based similarity used in all other experiments. We report on RMSE of total effect using *CMatch*_*MCL*_*C*__ and *CMatch*_*MCL*_*MSS*__ methods under contagion. Figure 14 shows that *CMatch*_*MCL*_*C*__ with L2-based similarity obtains the least error in all datasets except for Citeseer where Cosine similarity has a slightly lower error. For *CMatch*_*MCL*_*MSS*__, Cosine similarity has the lowest RMSE in Citeseer and 50 Women dataset, while Euclidean similarity has the lowest error in the other datasets. Jaccard similarity has the highest estimation error in all almost all cases.

## 8. Conclusion

In this paper, we proposed two different frameworks for network experiment designs that provide a more accurate estimation of two common causal estimands under interference: direct treatment effects and total treatment effects. For direct treatment effect estimation, we presented *CauseIS*, a framework that uses an independent set explicitly to disentangle peer effects from direct treatment effect estimation and increase the accuracy of direct treatment effect estimation. For total treatment effect estimation, we introduced *CMatch*, the first optimization framework that minimizes both interference and selection bias in cluster-based network experiment design. Our experiments on synthetic and real-world datasets confirm that this approach decreases direct and total treatment effect estimation error significantly. Some possible extensions of our frameworks include understanding the impact of network structural properties on estimation, jointly optimizing for interference and selection bias, and developing frameworks that are able to mitigate multiple-hop diffusions.

## Data availability statement

The original contributions presented in the study are included in the article/supplementary material, further inquiries can be directed to the corresponding author.

## Ethics statement

Ethical approval was not required for the study involving human data in accordance with the local legislation and institutional requirements. Written informed consent from the participants or their legal guardian/next of kin was not required in accordance with the national legislation and the institutional requirements.

## Author contributions

ZF and EZ contributed to the brainstorming, conception, and design of the study. ZF implemented the ideas and performed the statistical analysis under EZ's supervision. ZF wrote the first draft of the manuscript. All authors contributed to manuscript revision, read, and approved the submitted version.
